# Sociodemographic, general health and oral health factors associated with difficulty in eating among elderly people: a cross-sectional study, Brazil, 2019

**DOI:** 10.1590/S2237-96222025v34e20240678.en

**Published:** 2025-08-04

**Authors:** Laís Naara de Sousa Soares, Ana Estefanny Alves Cabral, Sabrina Barth de Andrade Luz, Mariella Agostinho Gonçalves Lourenço, Rafael Barroso Pazinatto, Bianca Souto de Medeiros Santos, Laércio Almeida de Melo

**Affiliations:** 1 Universidade Federal do Rio Grande do Norte, Departamento de Odontologia, Natal, RN, Brazil; 2 Universidade Federal de Juiz de Fora, Departamento de Odontologia Restauradora, Juiz de Fora, MG, Brazil

**Keywords:** Elderly, Diet, Prevalence, Oral Health, Cross-Sectional Studies, Anciano, Dieta, Prevalencia, Salud Bucal, Estudios Transversales

## Abstract

**Objective:**

To identify sociodemographic, general health, and oral health factors associated with difficulty in eating among elderly people in Brazil.

**Methods:**

This is a cross-sectional, population-based study that used data from the 2019 National Health Survey with elderly people (60+ years). Crude analysis was performed using the chi-square test to verify associations between variables and difficulty in eating. After investigating multicollinearity, adjusted prevalence ratios (PR) were calculated using Poisson multiple regression. Cramer’s V was used to measure effect size.

**Results:**

The sample consisted of 21,048 elderly individuals. Prevalence of severe or very severe difficulty in eating was 4.1% (95% confidence interval [95%CI] 3.7; 4.5). Multivariate analysis revealed that this condition was higher for females (PR 1.00; 95%CI 1.00; 1.01), older age (PR 1.01; 95%CI 1.00; 1.03), being illiterate (PR 1.01; 95%CI 1.00; 1.01), smoking (PR 1.01; 95%CI 1.00; 1.02), not having health insurance (PR 1.01; 95%CI 1.00; 1.01), multimorbidity (PR 1.01; 95%CI 1.01; 1.02), not brushing teeth daily (PR 1.07; 95%CI 1.03; 1.12) and not using dentures (PR 1.02; 95%CI 1.01; 1.02). Cramer’s V indicated that associations that were significant had a weak effect.

**Conclusion:**

Eating difficulties among the elderly were greater in those with unfavorable socioeconomic conditions, smokers, those with multimorbidity, those with inadequate oral hygiene and those who did not use dentures. The associations found suggest a weak relationship, indicating that other factors may also influence eating difficulties.

## Resumen

**Objetivo:**

Identificar los factores sociodemográficos, de salud general y de salud bucal asociados a la dificultad para alimentarse entre ancianos en Brasil.

**Métodos:**

Se trata de un estudio transversal, de base poblacional, que utilizó datos de la Encuesta Nacional de Salud 2019 con personas mayores (60+ años). Se realizó un análisis crudo mediante la prueba de chi-cuadrado para verificar asociaciones entre las variables y la dificultad para alimentarse. Después de investigar la multicolinealidad, se calcularon razones de prevalencia (RP) ajustadas mediante regresión múltiple de Poisson. Se utilizó el valor V de Cramer para evaluar el tamaño del efecto.

**Resultados:**

La muestra estuvo constituida por 21.048 personas mayores. La prevalencia de dificultad intensa o muy intensa para alimentarse fue del 4,1% (intervalo de confianza del 95% [IC95%] 3,7; 4,5). El análisis multivariado reveló que esta condición era mayor en mujeres (RP 1,00; IC95% 1,00; 1,01), mayor edad (RP 1,01; IC95% 1,00; 1,03), analfabetismo (RP 1,01; IC95% 1,00; 1,01), tabaquismo (RP 1,01; IC95% 1,00; 1,02), ausencia de seguro médico (RP 1,01; IC95% 1,00; 1,01), multimorbilidad (RP 1,01; IC95% 1,01; 1,02), no cepillarse los dientes a diario (RP 1,07; IC95% 1,03; 1,12) y no usar dentaduras postizas (RP 1,02; IC95% 1,01; 1,02). El valor V de Cramer indicó que las asociaciones significativas fueron de efecto bajo.

**Conclusión:**

Las dificultades alimentarias en los ancianos fueron mayores en aquellos con condiciones socioeconómicas desfavorables, fumadores, con multimorbilidad, con higiene bucal inadecuada y en aquellos que no usaban prótesis dental. Las asociaciones observadas sugieren una relación débil, lo que indica que otros factores también pueden influir en las dificultades de alimentación.

**Ethical aspects**

This research respected ethical principles, having obtained the following approval data:

Research Ethics Committee: National Research Ethics Committee

Opinion number: 3,529,376

Approval date: 23/8/2019

Informed Consent Form: Obtained from all participants prior to data collection.

## Introduction

Worldwide, the number of elderly people has increased significantly. This phenomenon is the result of several factors, such as the reduction in birth and death rates, in addition to the considerable increase in the average longevity of the population (1). With advances in medicine, human longevity has increased, which allows more people to live to old age. Improvements in living conditions and access to medical care have contributed to reduced mortality, especially among the elderly (2). This increase in the number of elderly people brings several challenges (3). The demographic transition is accompanied by high demands in several areas of health, including specialized care such as oral health and psychological care (4).

Aging results in the body undergoing anatomical and functional changes that affect the general health of the elderly. Such changes can affect the functional and metabolic capacities of these individuals. It is common for the elderly to have difficulty eating and, consequently, their ability to absorb nutrients is compromised (5-7). When seeking quality of life among the elderly, eating habits should be an important point to be considered, since the factors that interfere with nutrient intake are prominent risks for triggering malnutrition (8).

In addition to morphological changes resulting from the natural aging process, other causes also interfere with food intake, such as use of medications, chronic diseases, and socioeconomic and psychological factors (9). A further factor that influences the elderly population’s ability to eat is oral health (10). Most of the elderly population has oral health considered to be precarious (8).

Poor oral hygiene, associated with decreased manual dexterity caused by age, contributes to the onset of tooth decay, which is the main cause of declining oral health among the elderly, along with periodontal disease, use of poorly fitted dentures and xerostomia (9). The combination of these untreated problems results in greater loss of teeth, which causes difficulty chewing and the need to use dentures, which can predispose these individuals to malnutrition (11).

Given the negative impact that poor nutrition can have on the elderly population, this study aimed to identify sociodemographic, general health and oral health factors associated with difficulty in eating reported by the elderly. Identifying aspects that interfere with the nutritional status of this population will allow the development of interventions that minimize nutritional deficiencies and the worsening of the decline in the health and quality of life of the elderly.

## Methods

### Design

This is a population-based cross-sectional study. It was undertaken based on the most recent version of the National Health Survey, conducted in Brazil in 2019.

### Setting

The National Health Survey was a household survey conducted throughout Brazil. The survey was the result of a partnership between the Ministry of Health and the Brazilian Institute of Geography and Statistics, and is widely recognized as the gold standard for health surveys in Brazil. The survey analyzed a total of 90,846 individuals aged 15+.

### Participants

Only elderly individuals aged 60+ who reported having difficulty eating were assessed in this study. The National Health Survey sample is representative of the elderly population in Brazil. Details on the sample size calculation can be found in the methodology described in the National Health Survey (12).

### Variables

In order to identify individuals with eating difficulties, the elderly were asked to report their eating difficulties by answering the question: “What degree of difficulty do you have eating?” The answer to this question could be: “None”, “Mild”, “Severe” or “Very severe”. For data analysis, the variable was categorized as “None or slight difficulty” and “Severe or very severe”.

The variables of this study were collected using data available in the National Health Survey as self-reported by the elderly respondents. The socioeconomic and demographic variables were represented by sex, age, race/skin color, marital status, schooling, health insurance and dental insurance. Presence of schooling was defined based on the question about the ability to read and write, with those who answered “yes” being considered literate and those who answered “no” being considered illiterate.

The following variables related to general and oral health were analyzed: the habit of drinking alcoholic beverages (yes, no), the habit of smoking (yes, no), presence of multimorbidity (defined by the coexistence of two or more chronic diseases), tooth brushing frequency (classified as “does not brush every day” and ““brushes at least once a day”), complete edentulism (whether the elderly person had completely lost their teeth or not) and use of dentures (whether or not they used some type of denture). The chronic diseases considered for the multimorbidity variable were those analyzed by the National Health Survey, including hypertension, diabetes, high cholesterol, heart disease, stroke, asthma, rheumatism, back problems, musculoskeletal disorders, depression, schizophrenia, chronic lung disease, cancer and kidney failure.

### Data source and measurement 

This study used secondary data from the 2019 National Health Survey conducted in Brazil. The data source is available at: https://www.pns.icict.fiocruz.br/bases-de-dados/ (13).

### Bias control 

Bias control in this study was based on the National Health Survey sampling strategy, which took into account the distinct probabilities of selection and the characteristics of the complex sampling design. Sampling weights were defined for both the households and the residents selected, with the final weight resulting from the inverse of the probabilities of selection at each stage of the sampling plan. Adjustments for non-responses and corrections of population totals were included. In the data analysis, since the sample originated from a cluster survey, a specialized statistical analysis program was used, capable of considering the effects of data stratification and aggregation in the estimation of indicators and their precision measures.

### Study size

This was a nationwide study, carried out in all Brazilian macro-regions. The National Health Survey took into account households in urban and rural areas, state capitals and metropolitan regions.

### Statistical methods

The data were analyzed using the Statistical Package for the Social Sciences, version 22.0. Initially, the frequency distribution of all variables, both dependent and independent, was performed to create tables. In order to identify association between socioeconomic, oral health and general health variables and difficulty in eating, the crude prevalence ratios were calculated using the chi-square test between each variable and difficulty in eating, adopting a 95% confidence level.

Variables with p-values≤0,20 were considered for multicollinearity analysis. In order to investigate multicollinearity between these variables, an additional chi-square test was applied between them. This procedure sought to verify the existence of statistically significant associations that would indicate the possibility of the variables being excessively correlated, which could distort the results of the multivariate analysis.

Variables that showed a strong association in the multicollinearity test, defined by a p-value≤0,01, were considered multicollinear and excluded from the multivariate analysis. This strict criterion was adopted due to the large sample size, which could allow detection of more subtle associations between the variables and was in agreement with other large-scale samples (14,15).

For the multivariate analysis, adjusted prevalence ratios were calculated using Poisson multiple regression. Robust variance was used to correct for potential heteroscedasticity problems and to ensure that confidence intervals and p-values were adequately adjusted.

The Poisson multiple regression model was adjusted for all variables that did not present multicollinearity, using a 95% confidence level. In all phases of the analysis, the data were adjusted taking into account the impact of the sampling design, non-response rates and post-stratification weights. The null hypothesis of this study was that eating difficulties are not related to socioeconomic, demographic, general and oral health conditions.

Cramer’s V was calculated as an additional measure to assess the magnitude of association between the dependent and independent categorical variables, which indicated the effect size of the associations observed in the analyses.

## Results

This study analyzed 21,048 elderly individuals, with ages ranging from 60 to 107 years and a mean of 69.9 years (± 7.7). The descriptive analysis of the results showed that the majority of individuals analyzed were female (55.3%), 60 to 69 years old (55.9%), White (44.1%), married (44.2%), literate (77.2%), did not drink alcoholic beverages (74.7%), did not smoke (88.5%), did not have health insurance (73.5%), did not have dental insurance (91.9%), had two or more chronic diseases (57.4%), brushed their teeth at least once a day (99.5%), had not completely lost their teeth (68.9%) and used some type of prosthesis (74.3%).

Prevalence of elderly individuals with severe or very severe difficulty in eating due to oral problems was 4.1% (95% confidence interval [95%CI] 3.7; 4.5). As a result of the initial analysis and verification of multicollinearity between the variables, “race/skin color”, “marital status” and “alcoholic beverage” were not included in the Poisson multiple regression adjustment model due to their strong association with the other independent variables (Table 1).

**Table 1:** Crude prevalence ratio (PR) and crude 95% confidence interval (95%CI) for difficulty in eating according to socioeconomic, demographic, oral health and general health variables. Brazil, 2019 (n=21,048)

Variable	Severe or very severe difficulty (n=871)	None or slight difficulty (n=20,177)			
	n (%)	n (%)	PR (95%CI)	p-value	Cramer’s V
Sex^a^					
Female	511 (4.4)	11,131 (95.6)	1.40 (1.14; 1.71)	<0.001	0.014
Male	360 (3.8)	9,046 (96.2)			
**Age (years)^a^**					
60-69	400 (3.4)	11,360 (96.6)	1.00	0.002	0.054
70-79	291 (4.4)	6,312 (95.6)	1.01 (1.00; 1.02)		
≥80	180 (6.7)	2,505 (93.3)	1.02 (1.01; 1.04)		
**Race/skin color^a^**					
White	294 (3.2)	8,997 (96.8)	1.00	<0.001	0.047
Mixed race	439 (4.8)	8,741 (95.2)	1.02 (1.01; 1.02)		
Black	128 (5.7)	2,109 (94.3)	1.05 (1.03; 1.07)		
Other	10 (3.0)	328 (97.0)	0.99 (0.97; 1.02)		
Marital status^a^					
Single	198 (5.1)	3,652 (94.9)	1.00	0.003	0.039
Married	321 (3.5)	8,979 (96.5)	0.98 (0.97; 0.99)		
Divorced	80 (3.4)	2,248 (96.6)	0.99 (0.97; 1.00)		
Widowed	272 (4.9)	5,298 (95.1)	1.00 (0.98; 1.01)		
Schooling^a^					
Illiterate	346 (7.2)	4,455 (92.8)	2.34 (1.89; 2.88)	<0.001	0.084
Literate	525 (3.2)	15,722 (96.8)			
**Alcoholic beverage^a^**					
Yes	83 (2.3)	3,484 (97.7)	0.59 (0.44; 0.80)	<0.001	0.047
No	737 (4.7)	14,990 (95.3)			
**Tobacco smoking^a^**					
Yes	154 (6.3)	2,274 (93.7)	1.42 (1.09; 1.83)	0.011	0.040
No	717 (3.9)	17,903 (96.1)			
**Health insurance^a^**					
No	766 (4.9)	14,713 (95.1)	2.74 (2.05; 3.68)	<0.001	0.068
Yes	105 (1.9)	5,464 (98.1)			
Multimorbidity^a^					
Yes	601 (5.2)	10,954 (94.8)	2.39 (1.88; 3.05)	<0.001	0.069
No	212 (2.5)	8,377 (97.5)			
**Dental insurance^a^**					
No	836 (4.3)	18,505 (95.7)	2.27 (1.44; 3.57)	<0.001	0.031
Yes	35 (2.1)	1,672 (97.9)			
**Brushing frequency^a^**					
Does not brush every day	19 (17.3)	91 (82.7)	6.60 (3.91; 11.11)	<0.001	0.056
At least once a day	676 (3.4)	19,386 (96.6)			
**Complete edentulism^a^**					
Yes	370 (5.7)	6,168 (94.3)	1.76 (1.43; 2.17)	<0.001	0.051
No	501 (3.5)	14,009 (96.5)			
**Use of some kind of prosthesis^a^**					
No	419 (8.1)	4,752 (91.9)	2.15 (1.77; 2.60)	<0.001	0.110
Yes	448 (3.0)	14,535 (97.0)			

^a^The difference in relation to the total number of cases (100.0%) corresponds to the number of cases recorded as unknown, left blank or missing on the National Health Survey database.

In the multivariate analysis (Table 2), the variables considered were: sex, age, schooling, tobacco smoking, health insurance, multimorbidity, dental insurance, tooth brushing frequency, complete edentulism and use of some kind of prosthesis. We found that difficulty in eating was greater among elderly females (prevalence ratio [PR] 1.00; 95%CI 1.00; 1.01), the oldest elderly (PR 1.01; 95%CI 1.00; 1.03), those who were illiterate (PR 1.01; 95%CI 1.00; 1.01), smokers (PR 1.01; 95%CI 1.00; 1.02), those who did not have health insurance (PR 1.01; 95%CI 1.00; 1.01), those with multimorbidity (PR 1.01; 95%CI 1.01; 1.02), those who did not brush their teeth every day (PR 1.07; 95%CI 1.03; 1.12) and those who did not use dental prostheses (PR 1.02; 95%CI 1.01; 1.02).

**Table 2:** Adjusted prevalence ratio (PR) and adjusted 95% confidence interval (95%CI) for difficulty in eating according to socioeconomic, demographic, oral health and general health variables. Brazil, 2019 (n=21,048)

Variable	Severe or very severe difficulty (n=871)	None or slight difficulty (n=20,177)			
	n (%)	n (%)	Adjusted PR (Adjusted 95%CI)	p-value	Cramer’s V
Sex^a^					
Female	511 (4.4)	11,131 (95.6)	1.00 (1.00; 1.01)	0.010	0.014
Male	360 (3.8)	9,046 (96.2)			
**Age** (years)^a^					
60-69	400 (3.4)	11,360 (96.6)	1.00	<0.001	0.054
70-79	291 (4.4)	6,312 (95.6)	1.00 (1.00; 1.01)		
≥80	180 (6.7)	2,505 (93.3)	1.01 (1.00; 1.03)		
Schooling^a^					
Illiterate	346 (7.2)	4,455 (92.8)	1.01 (1.00; 1.01)	<0.001	0.084
Literate	525 (3.2)	15,722 (96.8)			
**Tobacco smoking^a^**					
Yes	154 (6.3)	2,274 (93.7)	1.01 (1.01 1.02)	<0.001	0.040
No	717 (3.9)	17,903 (96.1)			
**Health insurance^a^**					
No	766 (4.9)	14,713 (95.3)	1.01 (1.00; 1.01)	<0.001	0.068
Yes	105 (1.9)	5,464 (98.3)			
Multimorbidity^a^					
Yes	601 (5.2)	10,954 (94.8)	1.01 (1.01; 1.02)	<0.001	0.069
No	212 (2.5)	8,377 (97.5)			
**Dental insurance^a^**					
No	836 (4.3)	18,505 (95.7)	1.00 (0.99; 1.00)	0.773	0.031
Yes	35 (2.1)	1,672 (97.9)			
**Brushing frequency^a^**					
Does not brush every day	19 (17.3)	91 (82.7)	1.07 (1.03; 1.12)	0.002	0.056
At least once a day	676 (3.4)	19,386 (96.6)			
**Complete edentulism^a^**					
Yes	370 (5.7)	6,168 (94.3)	1.00 (1.00; 1.01)	0.251	0.051
No	501 (3.5)	14,009 (96.5)			
**Use of some kind of prosthesis^a^**					
No	419 (8.1)	4,752 (91.9)	1.02 (1.01 1.02)	<0.001	0.110
Yes	448 (3.0)	14,535 (97.0)			

^a^The difference in relation to the total number of cases (100.0%) corresponds to the number of cases recorded as unknown, left blank or missing on the National Health Survey database.

The effect size analysis, using Cramer’s V, revealed that the strength of the associations observed was weak. This indicated that, although the associations were statistically significant, they had limited impact in explaining the variation in eating difficulties among the elderly

## Discussion

The null hypothesis of this study was rejected. The results demonstrated that difficulty in eating is related to unfavorable socioeconomic conditions, unhealthy behaviors (such as smoking), accumulation of chronic diseases, poor oral hygiene and lack of use of dentures.

The statistical significance observed in the associations did not imply clinical or practical relevance, especially in studies with large samples, such as this study. Although a significant p-value indicates that association is unlikely to occur by chance, it does not guarantee that the observed effect is of large magnitude or has a relevant practical impact. Interpretation of statistical associations in large samples must consider the size of the effect, which is not always substantial, even if the p-value is low.

Analysis of Cramer’s V indicated that the associations observed between the variables and difficulty in eating were statistically significant, but with a weak effect. These variables explained part of the variation observed in eating difficulties, suggesting that other factors also influenced this condition. 

Despite some limitations, such as the cross-sectional nature of the data, which prevents determination of causal relationships, this study made important contributions to Brazilian reality. It showed that eating difficulties among the elderly can be mitigated by interventions that consider not only socioeconomic and behavioral factors, but also promotion of oral health, which includes adoption of adequate oral hygiene practices and use of dentures. The results of this study reinforce the need for a more integrated approach in health services, treating oral health as an important part of general care, essential for improving the diet and quality of life of the elderly.

Although the findings may guide public policies aimed at the health of older adults, especially the most vulnerable, such as those with low levels of education or multimorbidity, this study had limitations. The National Health Survey collected data on access to health services, including dental services, but these factors were not considered in this study, despite their possible influence on eating difficulties. Lack of territorial variables and factors such as social support limited the understanding of the broader determinants of this condition. Future studies could explore these issues, in addition to assessing the impact of regional disparities and access to health services in Brazil.

The association between difficulty in eating and being female observed in this study could be attributed to the greater longevity of women, which increases their exposure to chronic diseases, in addition to women having a higher prevalence of total tooth loss. Because women live longer, they tend to suffer more pronounced functional decline (16), which directly impacts their autonomy to feed themselves, making them more dependent. Tooth loss impairs biting and grinding of food, as it makes the chewing process difficult and impacts the ability to ingest nutritious foods.

Older elderly people had a significantly higher prevalence of eating difficulties. This was due to limitations that affect their ability to perform general daily activities. As they age, these individuals become more susceptible to chronic diseases, which worsen eating difficulties (17) and compromise quality of life. The act of eating in a nutritious and enjoyable way becomes more challenging.

Schooling was also shown to be a critical factor, as illiterate elderly individuals had greater eating difficulties. This result reflected reduced access to health information and preventive practices. It has been considered that illiterate elderly individuals are probably more susceptible to poorer general health conditions and poorer oral conditions throughout their lives. Such accumulated conditions contribute to high functional decline, compromised autonomy and a high number of tooth losses, which favors eating difficulties (1).

Tobacco smoking has been linked to tooth loss, which worsens chewing difficulties and limits the intake of nutritious foods (18). The effect of smoking is related to the development of periodontal diseases, which accelerate tooth loss, compromising chewing ability. The impact of smoking on general health increases the risk of chronic diseases, such as cardiovascular and respiratory diseases (19), and can lead to conditions that reduce the autonomy of older adults to perform daily activities, including eating. The significant relationship between smoking and eating difficulties reinforces the need for integrated health approaches that include tobacco control as part of oral health promotion in older adults.

Multimorbidity has been shown to be associated with greater eating difficulties among the elderly. It has been highlighted that chronic conditions, such as diabetes and hypertension, often affect oral health and worsen tooth loss and difficulty in eating (20). The presence of multiple diseases, in addition to impacting oral health, can also limit mobility and the ability of the elderly to maintain adequate oral hygiene, contributing to the worsening of oral conditions. Accumulation of chronic diseases is linked to functional decline, which can compromise the ability of the elderly to chew and swallow food. This combination of factors contributes significantly to eating difficulties.

Inadequate toothbrushing frequency suggests that poor oral hygiene may exacerbate existing problems, such as caries lesions and periodontal disease, and further compromise masticatory function. The presence of these diseases can cause pain and discomfort, which directly affects the ability to eat properly. Inadequate oral hygiene practices are common among elderly Brazilians, reflecting poorer oral health outcomes and, consequently, greater difficulty in performing daily activities, such as eating (21).

Absence of dentures was higher in those with higher prevalence of eating difficulties. Use of dentures is essential for restoring masticatory function in edentulous elderly individuals (those who have lost their natural teeth), promoting a more varied and nutritious diet (22). Rehabilitation through use of dentures improves masticatory efficiency and eating performance and allows for better biting and grinding of food. Edentulism without dental replacement causes not only functional limitations and eating difficulties, but also psychological harm, such as low self-esteem and social isolation, which can also reduce motivation for adequate nutrition (23).

Brushing frequency, edentulism and use of dentures are closely related, and this relationship may influence interpretation of the results. Edentulous individuals may not have been adequately instructed on oral hygiene care or may not brush their teeth in the same way as individuals who have teeth. Absence of dentures, associated with greater difficulty in eating observed in this study, may reflect the unmet need for dentures due to tooth loss, since those who do not use dentures do not always have all their natural teeth. 

The brushing frequency variable may not be completely representative of edentulous or denture-wearing elderly individuals, since these individuals may have different oral care practices. For a more accurate analysis, it would be important to consider these interrelationships, and future research could investigate, in more detail, the relationship between use of dentures, oral hygiene and eating difficulties, separating groups of denture-wearing and edentulous individuals, in order to better clarify the factors that influence the nutritional health of the elderly.

Although there were statistically significant associations between severe or very severe eating difficulty and several variables (Table 2), it is noteworthy that, for the sex, age, schooling, health insurance and complete edentulism variables, the confidence intervals of the adjusted prevalence ratios overlap with the null value (PR 1). This suggests greater uncertainty about the real magnitude of these associations. Despite the statistical significance, this overlap indicates that the effect estimates may be less precise and robust than the p values indicate. The associations observed are relevant, but caution is recommended when interpreting the results, especially for these specific variables.

In conclusion, the difficulty in eating among the elderly was greater in those with poorer socioeconomic conditions, those who smoke, those with a history of chronic diseases, those who neglect oral hygiene and those who do not use dentures. We recognize that these factors explain only part of the variation observed in eating difficulties, since other determinants must also be considered in intervention strategies and public policies aimed at the health of the elderly.

## Data Availability

The database, containing only the elderly population, used in the 2019 National Health Survey, is available at: https://drive.google.com/file/d/1VDjasU9Wrteyqfme5LmeCIAX45Z0QrXs/view?usp=sharing.
