# Supplementary material for: Sociodemographic, general health and oral health factors associated with difficulty in eating among elderly people: a cross-sectional study, Brazil, 2019
Source: Epidemiol Serv Saude. 2025 Aug 4;34:e20240678. doi: 10.1590/S2237-96222025v34e20240678.en (PMC12334233; doi:10.1590/S2237-96222025v34e20240678.en)
Supplement: Translation

# Translation: Fatores sociodemográficos de saúde geral e bucal associados à dificuldade de se alimentar em idosos: estudo transversal, Brasil, 2019

**DOI:** 10.1590/S2237-96222025v34e20240678.pt

**Authors:** Laís Naara de Sousa Soares, Ana Estefanny Alves Cabral, Sabrina Barth de Andrade Luz, Mariella Agostinho Gonçalves Lourenço, Rafael Barroso Pazinatto, Bianca Souto de Medeiros Santos, Laércio Almeida de Melo

**Affiliations:** 1 Universidade Federal do Rio Grande do Norte, Departamento de Odontologia, Natal, RN, Brasil; 2 Universidade Federal de Juiz de Fora, Departamento de Odontologia Restauradora, Juiz de Fora, MG, Brasil

**Keywords:** Idoso, Dieta, Prevalência, Saúde Bucal, Estudos Transversais

## Abstract

**Objetivo:**

Identificar os fatores sociodemográficos, de saúde geral e bucal associados à dificuldade de se alimentar em idosos no Brasil.

**Métodos:**

Trata-se de estudo transversal, de base populacional, que utilizou dados da Pesquisa Nacional de Saúde de 2019 com idosos (60+ anos). Foi realizada análise bruta por meio do teste qui-quadrado para verificar associações entre as variáveis e a dificuldade de se alimentar. Após investigação de multicolinearidade, as razões de prevalência (RP) ajustadas foram calculadas por regressão múltipla de Poisson. O valor de V de Cramer foi utilizado para avaliar o tamanho do efeito.

**Resultados:**

A amostra foi de 21.048 idosos. A prevalência de dificuldade intensa ou muito intensa de se alimentar foi 4,1% (intervalo de confiança de 95% [IC95%] 3,7; 4,5). A análise multivariada revelou que essa condição foi maior para sexo feminino (RP 1,00; IC95% 1,00; 1,01), maior idade (RP 1,01; IC95% 1,00; 1,03), não alfabetizados (RP 1,01; IC95% 1,00; 1,01), tabagismo (RP 1,01; IC95% 1,00; 1,02), ausência de plano de saúde (RP 1,01; IC95% 1,00; 1,01), multimorbidade (RP 1,01; IC95% 1,01; 1,02), não escovação diária (RP 1,07; IC95% 1,03; 1,12) e não uso de próteses dentárias (RP 1,02; IC95% 1,01; 1,02). O valor de V de Cramer indicou que as associações significativas foram de efeito fraco.

**Conclusão:**

A dificuldade alimentar em idosos foi maior naqueles com condições socioeconômicas desfavoráveis, fumantes, com multimorbidade, com higiene bucal inadequada e nos que não usavam próteses dentárias. As associações observadas sugerem relação fraca, indicando que outros fatores também podem influenciar a dificuldade alimentar.

**Aspectos éticos**

A presente pesquisa respeitou os princípios éticos, obtendo os seguintes dados de aprovação: :

Comitê de ética em pesquisa: Comissão Nacional de Ética em Pesquisa

Número do parecer: 3.529.376

Data de aprovação: 23/8/2019

Registro do consentimento livre e esclarecido: Obtido de todos os participantes antes da coleta.

## Introdução

No âmbito mundial, o número de pessoas idosas tem aumentado de maneira acentuada. Esse fenômeno é fruto de diversos fatores, como a redução nas taxas de natalidade e mortalidade, além do considerável aumento na longevidade média da população (1). Com os avanços na medicina, a longevidade humana tem se ampliado, o que permite que mais pessoas vivam até idades avançadas. Melhorias nas condições de vida e acesso a cuidados médicos têm contribuído para a redução da mortalidade, especialmente entre os mais velhos (2). Esse aumento no número de pessoas idosas traz consigo vários desafios (3). A transição demográfica é acompanhada por altas demandas em diversas áreas de saúde, incluindo cuidados especializados como a saúde bucal e a psicológica (4).

Com o avanço da idade, o organismo sofre alterações anatômicas e funcionais, que interferem nas condições gerais de saúde dos idosos. Tais mudanças podem afetar as capacidades funcionais e metabólicas desses indivíduos. É comum que os idosos possuam dificuldade de se alimentar e, consequentemente, a capacidade de absorção de nutrientes fica comprometida (5-7). Ao buscar qualidade de vida em idosos, os hábitos alimentares devem ser um ponto de importante observação, visto que os fatores que interferem nessa ingestão de nutrientes são riscos proeminentes para o desencadeamento da má nutrição (8).

Além das alterações morfológicas derivadas do processo natural de envelhecimento, outras causas também interferem na ingestão de alimentos, como o uso de medicamentos, as doenças crônicas e os fatores socioeconômicos e psicológicos (9). Outro fator que influencia na capacidade de alimentação da população idosa é a saúde bucal (10). A maior parte da população idosa possui a saúde bucal considerada como precária (8). 

A falta de higiene bucal, associada à diminuição de destreza manual causada pela idade, contribui para o surgimento da cárie dentária, principal responsável pelo declínio da saúde bucal nos idosos, juntamente com a doença periodontal, o uso de próteses mal ajustadas e a xerostomia (9). A combinação desses problemas não tratados resulta na maior perda de elementos dentários, o que ocasiona a dificuldade de mastigação e a necessidade do uso de próteses, que podem predispor esses indivíduos à má nutrição (11). 

Diante do impacto negativo que a má nutrição pode trazer à população idosa, este estudo objetivou identificar os fatores sociodemográficos, de saúde geral e bucal associados à dificuldade de se alimentar relatada por idosos. A identificação dos aspectos que interferem no estado nutricional dessa população permitirá a elaboração de intervenções que minimizem o déficit nutricional e o agravamento do declínio da saúde e qualidade de vida dos idosos. 

## Métodos

### Delineamento do estudo

Este trabalho é classificado como pesquisa transversal de base populacional. Para a execução deste, foi utilizada a versão mais recente da Pesquisa Nacional de Saúde, conduzida no Brasil em 2019.

### Contexto

A Pesquisa Nacional de Saúde foi conduzida por meio de inquérito domiciliar realizado em todo o território brasileiro. Tal pesquisa foi resultado da parceria entre o Ministério da Saúde e o Instituto Brasileiro de Geografia e Estatística, sendo amplamente reconhecida como o padrão-ouro dos inquéritos de saúde no país. Foram analisados 90.846 indivíduos com 15+ anos.

### Participantes

Foram avaliados, exclusivamente, idosos com 60+ anos que informaram ter dificuldade de se alimentar. A amostra da Pesquisa Nacional de Saúde é representativa para a população idosa brasileira. Os detalhes sobre o cálculo amostral podem ser consultados na metodologia descrita na Pesquisa Nacional de Saúde (12). 

### Variáveis

Para identificar os indivíduos com dificuldades de se alimentar, os idosos tinham que relatar a dificuldade de se alimentar a partir do questionamento: “Que grau de dificuldade o(a) Sr(a) tem para se alimentar?”. Esse questionamento poderia ter como resposta: “Nenhuma”, “Leve”, “Intensa” ou “Muito intensa”. Para a análise dos dados, a variável foi categorizada em “Nenhuma ou leve dificuldade” e “Intensa ou muito intensa”.

As variáveis deste estudo foram coletadas a partir do autorrelato dos idosos, utilizando os dados disponíveis na Pesquisa Nacional de Saúde. As variáveis socioeconômicas e demográficas foram representadas por sexo, idade, raça/cor da pele, estado civil, escolaridade, plano de saúde e plano odontológico. A escolaridade foi obtida com base na pergunta sobre a habilidade de ler e escrever, sendo considerados alfabetizados aqueles que responderam “sim” e não alfabetizados os que responderam “não”. 

No que se refere às variáveis relacionadas à saúde geral e bucal, foram analisados: o hábito de ingerir bebida alcoólica (sim, não), o hábito de fumar (sim, não), a presença de multimorbidade (definida pela coexistência de duas ou mais doenças crônicas), a frequência de escovação (classificada como “não escova todos os dias” e “escova pelo menos uma vez ao dia”), o edentulismo total (se o idoso perdeu completamente os dentes ou não) e o uso de prótese dentária (se utilizava ou não algum tipo de prótese). As doenças crônicas consideradas para a variável multimorbidade foram aquelas analisadas pela Pesquisa Nacional de Saúde, incluindo hipertensão, diabetes, colesterol alto, doenças cardíacas, acidente vascular cerebral, asma, reumatismo, problemas de coluna, distúrbios osteomusculares, depressão, esquizofrenia, doenças pulmonares crônicas, câncer e insuficiência renal.

### Fonte de dados e mensuração

Este estudo utilizou dados secundários da Pesquisa Nacional de Saúde de 2019, realizada no Brasil. A fonte de dados está disponível em: https://www.pns.icict.fiocruz.br/bases-de-dados/ (13).

### Controle de viés

O controle de viés neste estudo foi baseado na estratégia de amostragem da Pesquisa Nacional de Saúde, que levou em consideração as probabilidades distintas de seleção e as características do desenho amostral complexo. Foram definidos pesos amostrais tanto para os domicílios quanto para os moradores selecionados, com o peso final resultando do inverso das probabilidades de seleção em cada estágio do plano amostral. Incluíram-se ajustes para não respostas e correções dos totais populacionais. Na análise dos dados, como a amostra se originou de pesquisa por conglomerados, foi utilizado um programa de análise estatística especializado, capaz de considerar os efeitos da estratificação e da agregação dos dados na estimação dos indicadores e suas medidas de precisão.

### Tamanho do estudo

Tratou-se de estudo de abrangência nacional, realizado em todas as macrorregiões brasileiras. A Pesquisa Nacional de Saúde levou em consideração domicílios em áreas urbanas e rurais, capitais e regiões metropolitanas.

### Métodos estatísticos

Os dados foram analisados por meio do software Statistical Package for the Social Science, versão 22.0. Inicialmente, procedeu-se à distribuição de frequência de todas as variáveis, tanto dependentes quanto independentes, visando à elaboração de tabelas. Com o propósito de identificar a associação entre as variáveis socioeconômicas, de saúde bucal e de saúde geral e a dificuldade de se alimentar, foram calculadas as razões de prevalência bruta através do teste qui-quadrado entre cada variável e a dificuldade de se alimentar, adotando o nível de confiança de 95%. 

As variáveis com p-valor≤0,20 foram consideradas para a análise de multicolinearidade. Para investigar a multicolinearidade entre essas variáveis, foi aplicado o teste qui-quadrado adicional entre elas. Esse procedimento buscou verificar a existência de associações estatisticamente significativas que indicariam a possibilidade de as variáveis estarem correlacionadas de forma excessiva, o que poderia distorcer os resultados da análise multivariada.

As variáveis que apresentaram associação forte no teste de multicolinearidade, definida por p-valor≤0,01, foram consideradas como multicolineares e excluídas da análise multivariada. Esse critério rigoroso foi adotado devido ao grande tamanho da amostra, o que pôde permitir a detecção de associações mais sutis entre as variáveis e estava de acordo com outras amostras de grande proporção (14,15).

Para a análise multivariada, as razões de prevalência ajustadas foram calculadas utilizando a regressão múltipla de Poisson. A utilização da variância robusta foi adotada para corrigir possíveis problemas de heterocedasticidade e garantir que os intervalos de confiança e p-valores fossem adequadamente ajustados.

O modelo de regressão múltipla de Poisson foi ajustado para todas as variáveis que não apresentaram multicolinearidade, utilizando o nível de confiança de 95%. Em todas as fases da análise, os dados foram ajustados levando em conta o impacto do desenho amostral, as taxas de não resposta e os pesos de pós-estratificação. A hipótese nula deste estudo é de que a dificuldade de se alimentar não está relacionada com condições socioeconômicas, demográficas, de saúde geral e bucal.

O V de Cramer foi calculado como medida adicional para avaliar a magnitude da associação entre as variáveis categóricas dependentes e independentes, o que indicou o tamanho do efeito das associações observadas nas análises.

## Resultados

Neste estudo, 21.048 idosos foram analisados, com idades variando de 60-107 anos e média de 69,9 anos (±7,7). A análise descritiva dos resultados evidenciou que a maioria dos indivíduos analisados é do sexo feminino (55,3%), com 60-69 anos (55,9%), de raça/cor da pele branca (44,1%), casados (44,2%), alfabetizados (77,2%), que não fazem uso de bebida alcoólica (74,7%), que não fumam (88,5%) sem plano de saúde (73,5%), sem plano odontológico (91,9%), portadores de duas ou mais doenças crônicas (57,4%), que escovam os dentes pelo menos uma vez ao dia (99,5%), que não perderam totalmente seus dentes (68,9%) e que fazem uso de algum tipo de prótese (74,3%). 

A prevalência de idosos com dificuldade intensa ou muito intensa para se alimentar devido a problemas bucais foi 4,1% (intervalo de confiança de 95% [IC95%] 3,7; 4,5). Como resultado da análise inicial e da verificação da multicolinearidade entre as variáveis, a “raça/cor da pele”, “estado civil” e “bebida alcoólica” não foram incluídos no modelo de ajuste da regressão múltipla de Poisson devido à forte associação com as outras variáveis independentes (Tabela 1). Na análise multivariada (Tabela 2), as variáveis consideradas foram: sexo, idade, escolaridade, fumo, plano de saúde, multimorbidade, plano odontológico, frequência de escovação, edentulismo total e uso de algum tipo de prótese. Verificou-se que a dificuldade de se alimentar foi maior nos idosos do sexo feminino (razão de prevalência [RP] 1,00; IC95% 1,00; 1,01), nos mais velhos (RP 1,01; IC95% 1,00; 1,03), nos não alfabetizados (RP 1,01; IC95% 1,01 ;1,00 %95), naqueles que fumam (RP 1,01; IC95% 1,00; 1,02), nos que não possuem plano de saúde (RP 1,01; IC95% 1,00; 1,01), nos que possuem multimorbidade (RP 1,01; IC95% 1,01; 1,02), naqueles que não escovam os dentes todos os dias (RP 1,07; IC95% 1,03; 1,12) e nos que não fazem uso de próteses dentárias (RP 1,02; IC95% 1,01; 1,02).

**Tabela 1:** Razão de prevalência (RP) bruta e intervalo de confiança de 95% (IC95%) bruto da dificuldade de se alimentar pelas variáveis socioeconômicas, demográficas, de saúde bucal e geral. Brasil, 2019 (n=21.048)

Variável	Dificuldade intensa ou muito intensa (n=871)	Nenhuma ou leve dificuldade (n=20.177)			
	n (%)	n (%)	RP (IC95%)	p-valor	V de Cramer
Sexo^a^					
Feminino	511 (4,4)	11.131 (95,6)	1,40 (1,14; 1,71)	<0,001	0,014
Masculino	360 (3,8)	9.046 (96,2)			
**Idade (anos)^a^**					
60-69	400 (3,4)	11.360 (96,6)	1,00	0,002	0,054
70-79	291 (4,4)	6.312 (95,6)	1,01 (1,00; 1,02)		
≥80	180 (6,7)	2.505 (93,3)	1,02 (1,01; 1,04)		
**Raça/cor da pele^a^**					
Branco	294 (3,2)	8.997 (96,8)	1,00	<0,001	0,047
Pardo	439 (4,8)	8.741 (95,2)	1,02 (1,01; 1,02)		
Preto	128 (5,7)	2.109 (94,3)	1,05 (1,03; 1,07)		
Outros	10 (3,0)	328 (97,0)	0,99 (0,97; 1,02)		
**Estado civil^a^**					
Solteiro	198 (5,1)	3.652 (94,9)	1,00	0,003	0,039
Casado	321 (3,5)	8.979 (96,5)	0,98 (0,97; 0,99)		
Divorciado	80 (3,4)	2.248 (96,6)	0,99 (0,97; 1,00)		
Viúvo	272 (4,9)	5.298 (95,1)	1,00 (0,98; 1,01)		
Escolaridade^a^					
Não alfabetizado	346 (7,2)	4.455 (92,8)	2,34 (1,89; 2,88)	<0,001	0,084
Alfabetizado	525 (3,2)	15.722 (96,8)			
**Bebida alcoólica^a^**					
Sim	83 (2,3)	3.484 (97,7)	0,59 (0,44; 0,80)	<0,001	0,047
Não	737 (4,7)	14.990 (95,3)			
Fumo^a^					
Sim	154 (6,3)	2.274 (93,7)	1,42 (1,09; 1,83)	0,011	0,040
Não	717 (3,9)	17.903 (96,1)			
**Plano de saúde^a^**					
Não	766 (4,9)	14.713 (95,1)	2,74 (2,05; 3,68)	<0,001	0,068
Sim	105 (1,9)	5.464 (98,1)			
Multimorbidade^a^					
Sim	601 (5,2)	10.954 (94,8)	2,39 (1,88; 3,05)	<0,001	0,069
Não	212 (2,5)	8.377 (97,5)			
**Plano odontológico^a^**					
Não	836 (4,3)	18.505 (95,7)	2,27 (1,44; 3,57)	<0,001	0,031
Sim	35 (2,1)	1.672 (97,9)			
**Frequência de escovação^a^**					
Não escova todos os dias	19 (17,3)	91 (82,7)	6,60 (3,91; 11,11)	<0,001	0,056
Pelo menos uma vez ao dia	676 (3,4)	19.386 (96,6)			
**Edentulismo total^a^**					
Sim	370 (5,7)	6.168 (94,3)	1,76 (1,43; 2,17)	<0,001	0,051
Não	501 (3,5)	14.009 (96,5)			
**Uso de algum tipo de prótese^a^**					
Não	419 (8,1)	4.752 (91,9)	2,15 (1,77; 2,60)	<0,001	0,110
Sim	448 (3,0)	14.535 (97,0)			

^a^A diferença para o número total de casos (100,0%) corresponde ao número de casos ignorados, em branco ou com preenchimento ausente no banco de dados da Pesquisa Nacional de Saúde.

**Tabela 2:** Razão de prevalência (RP) ajustada e intervalo de confiança de 95% (IC95%) ajustado da dificuldade de se alimentar pelas variáveis socioeconômicas, demográficas, de saúde bucal e geral. Brasil, 2019 (n=21.048)

Variável	Dificuldade intensa ou muito intensa (n=871)	Nenhuma ou leve dificuldade (n=20.177)			
	n (%)	n (%)	RP ajustada (IC95% ajustado)	p-valor	V de Cramer
Sexo^a^					
Feminino	511 (4,4)	11.131 (95,6)	1,00 (1,00; 1,01)	0,010	0,014
Masculino	360 (3,8)	9.046 (96,2)			
**Idade (anos)^a^**					
60-69	400 (3,4)	11.360 (96,6)	1,00	<0,001	0,054
70-79	291 (4,4)	6.312 (95,6)	1,00 (1,00; 1,01)		
≥80	180 (6,7)	2.505 (93,3)	1,01 (1,00-1,03)		
Escolaridade^a^					
Não alfabetizado	346 (7,2)	4.455 (92,8)	1,01 (1,00; 1,01)	<0,001	0,084
Alfabetizado	525 (3,2)	15.722 (96,8)			
Fumo^a^					
Sim	154 (6,3)	2.274 (93,7)	1,01 (1,01; 1,02)	<0,001	0,040
Não	717 (3,9)	17.903 (96,1)			
**Plano de saúde^a^**					
Não	766 (4,9)	14.713 (95,3)	1,01 (1,00; 1,01)	<0,001	0,068
Sim	105 (1,9)	5.464 (98,3)			
Multimorbidade^a^					
Sim	601 (5,2)	10.954 (94,8)	1,01 (1,01; 1,02)	<0,001	0,069
Não	212 (2,5)	8.377 (97,5)			
**Plano odontológico^a^**					
Não	836 (4,3)	18.505 (95,7)	1,00 (0,99; 1,00)	0,773	0,031
Sim	35 (2,1)	1.672 (97,9)			
**Frequência de escovação^a^**					
Não escova todos os dias	19 (17,3)	91 (82,7)	1,07 (1,03; 1,12)	0,002	0,056
Pelo menos uma vez ao dia	676 (3,4)	19.386 (96,6)			
**Edentulismo total^a^**					
Sim	370 (5,7)	6.168 (94,3)	1,00 (1,00; 1,01)	0,251	0,051
Não	501 (3,5)	14.009 (96,5)			
**Uso de algum tipo de prótese^a^**					
Não	419 (8,1)	4.752 (91,9)	1,02 (1,01; 1,02)	<0,001	0,110
Sim	448 (3,0)	14.535 (97,0)			

^a^A diferença para o número total de casos (100,0%) corresponde ao número de casos ignorados, em branco ou com preenchimento ausente no banco de dados da Pesquisa Nacional de Saúde.

A análise do tamanho do efeito, através do valor de V de Cramer, revelou que a força das associações observadas foi fraca. Isso indicou que, embora as associações sejam estatisticamente significativas, elas possuem impacto limitado na explicação da variação da dificuldade de se alimentar entre os idosos. 

## Discussão

A hipótese nula deste estudo foi rejeitada. Os resultados demonstraram que a dificuldade de se alimentar está relacionada com condições socioeconômicas desfavoráveis, comportamentos prejudiciais à saúde (como o tabagismo), acúmulo de doenças crônicas, falta de higiene bucal e falta de uso de próteses dentárias.

A significância estatística observada nas associações não implicou relevância clínica ou prática, especialmente em estudos com grandes amostras, como este estudo. Embora um p-valor significativo indique que a associação é improvável de ocorrer por acaso, isso não garante que o efeito observado seja de grande magnitude ou tenha impacto prático relevante. A interpretação de associações estatísticas em grandes amostras deve considerar o tamanho do efeito, que nem sempre é substancial, mesmo que o p-valor seja baixo. 

A análise do valor de V de Cramer indicou que as associações observadas entre as variáveis e a dificuldade de se alimentar foram estatisticamente significativas, mas de efeito fraco. Essas variáveis explicaram parte da variação observada na dificuldade alimentar, sugerindo que outros fatores também influenciavam essa condição.

Apesar de algumas limitações, como a natureza transversal dos dados, que impede a determinação de relações causais, este estudo trouxe importantes contribuições para a realidade brasileira. Evidenciou-se que as dificuldades alimentares entre idosos podem ser atenuadas por intervenções que considerem não apenas fatores socioeconômicos e comportamentais, mas também a promoção da saúde bucal, o que inclui a adoção de práticas adequadas de higiene bucal e o uso de próteses dentárias. A pesquisa reforçou a necessidade da abordagem mais integrada nos serviços de saúde, tratando a saúde bucal como parte importante do cuidado geral, essencial para melhorar a alimentação e a qualidade de vida dos idosos. 

Embora os achados possam orientar políticas públicas voltadas para a saúde dos idosos, especialmente os mais vulneráveis, como os com baixo nível de escolaridade ou multimorbidade, este estudo apresentou limitações. A Pesquisa Nacional de Saúde coletou dados sobre o acesso a serviços de saúde, incluindo os odontológicos, porém esses fatores não foram considerados neste estudo, apesar de sua possível influência sobre as dificuldades alimentares. A falta de variáveis territoriais e de fatores como apoio social limitou a compreensão dos determinantes mais amplos dessa condição. Futuros estudos poderiam explorar essas questões, além de avaliar o impacto das disparidades regionais e o acesso a serviços de saúde no Brasil.

A associação entre dificuldade de se alimentar e o sexo feminino observada neste estudo pôde ser atribuída à maior longevidade das mulheres, o que aumenta a exposição a doenças crônicas, além de as mulheres apresentarem maior prevalência de perda dentária total. As mulheres, por viverem mais, tendem a sofrer declínio funcional mais acentuado (16), o que impacta diretamente na autonomia para se alimentar sozinhas, deixando-as mais dependentes. A perda dentária prejudica a apreensão e a trituração dos alimentos, pois dificulta o processo de mastigação e impacta a capacidade de ingestão de alimentos nutritivos. 

Os idosos em faixas etárias mais avançadas apresentaram prevalência significativamente maior de dificuldades alimentares. Isso ocorreu devido às limitações que afetam sua capacidade de realizar atividades diárias gerais. À medida que envelhecem, esses indivíduos se tornam mais susceptíveis a doenças crônicas, que agravam as dificuldades alimentares (17) e comprometem a qualidade de vida. O ato de se alimentar de maneira nutritiva e prazerosa torna-se mais desafiador.

A escolaridade também se mostrou como fator crítico, pois idosos não alfabetizados apresentavam maior dificuldade para se alimentar. Esse resultado refletiu o menor acesso a informações de saúde e práticas preventivas. Considerou-se que, provavelmente, os idosos analfabetos estão mais susceptíveis a piores condições de saúde geral e a piores condições bucais ao longo da vida. Tais condições acumuladas contribuem para o alto declínio funcional, o comprometimento de autonomia e o número elevado de perdas dentárias, o que favorece dificuldades na alimentação (1).

O tabagismo foi relacionado à perda dentária, o que agrava as dificuldades mastigatórias e limita a ingestão de alimentos nutritivos (18). O efeito do tabagismo está relacionado ao desenvolvimento de doenças periodontais, que aceleram a perda dentária, comprometendo a capacidade mastigatória. O impacto do tabagismo na saúde geral aumenta o risco de doenças crônicas, como doenças cardiovasculares e respiratórias (19), e pode levar a condições que reduzem a autonomia do idoso para realizar atividades cotidianas, incluindo a alimentação. A relação significativa entre tabagismo e dificuldade de alimentação reforça a necessidade de abordagens integradas de saúde que incluam o controle do tabagismo como parte da promoção da saúde bucal em idosos.

A multimorbidade mostrou-se associada a maiores dificuldades alimentares em idosos. Destacou-se que condições crônicas, como diabetes e hipertensão, frequentemente afetam a saúde bucal e agravam a perda de dentes e a dificuldade de se alimentar (20). A presença de múltiplas doenças, além de impactar a saúde bucal, também pode limitar a mobilidade e a capacidade dos idosos em manter a higiene bucal adequada, contribuindo para o agravamento das condições bucais. O acúmulo de doenças crônicas está ligado ao declínio funcional, o que pode comprometer a capacidade dos idosos de mastigar e engolir os alimentos. Essa combinação de fatores contribui significativamente para a dificuldade alimentar.

A frequência inadequada de escovação dental sugere que a manutenção deficiente da higiene bucal pode exacerbar problemas já existentes, como lesões de cáries e doenças periodontais, e comprometer ainda mais a função mastigatória. A presença dessas doenças pode causar dor e desconforto, o que afeta diretamente a capacidade de se alimentar de forma adequada. Práticas inadequadas de higiene bucal são comuns entre os idosos brasileiros, refletindo piores desfechos de saúde bucal e, consequentemente, maior dificuldade de realizar atividades cotidianas, como a alimentação (21).

A ausência de próteses dentárias foi maior naqueles com maior prevalência de dificuldade de alimentação. O uso de próteses dentárias é essencial para restaurar a função mastigatória em idosos edêntulos (que perderam seus dentes naturais), promovendo uma dieta mais variada e nutritiva (22). A reabilitação por meio do uso de próteses dentárias melhora a eficiência mastigatória e a performance alimentar e possibilita a melhor apreensão e trituração dos alimentos. O edentulismo sem reposição dentária acarreta não apenas limitações funcionais e dificuldade na alimentação, mas também prejuízos psicológicos, como baixa autoestima e isolamento social, que também podem reduzir a motivação para a alimentação adequada (23). 

A frequência de escovação, o edentulismo e o uso de prótese dentária estão intimamente relacionados, e essa relação pode influenciar a interpretação dos resultados. Indivíduos edêntulos podem não ter sido adequadamente instruídos sobre os cuidados de higiene bucal ou podem não realizar escovação da mesma forma que indivíduos dentados. A ausência de prótese dentária, associada à maior dificuldade alimentar observada neste estudo, pode refletir a necessidade não atendida de prótese devido à perda dentária, já que quem não utiliza prótese nem sempre possui todos os dentes naturais. 

A variável frequência de escovação pode não ser completamente representativa para idosos edêntulos ou usuários de prótese, uma vez que esses indivíduos podem ter cuidados bucais diferenciados. Para uma análise mais precisa, seria importante considerar essas inter-relações, e futuras pesquisas poderiam investigar, de forma mais detalhada, a relação entre o uso de prótese, a higiene bucal e a dificuldade alimentar, separando grupos de dentados e edêntulos, a fim de esclarecer melhor os fatores que influenciam a saúde alimentar dos idosos.

Embora existam associações estatisticamente significativas entre a dificuldade intensa ou muito intensa de alimentação e diversas variáveis (Tabela 2), destaca-se que, para as variáveis sexo, idade, escolaridade, plano de saúde e edentulismo total, os intervalos de confiança das razões de prevalência ajustadas se sobrepõem ao valor nulo (RP 1). Isso sugere maior incerteza sobre a real magnitude dessas associações. Apesar da significância estatística, essa sobreposição indica que as estimativas de efeito podem ser menos precisas e robustas do que os valores de p indicam. As associações observadas são relevantes, mas recomenda-se cautela na interpretação dos resultados, principalmente para essas variáveis específicas.

Como conclusão, a dificuldade de se alimentar em idosos foi maior naqueles com piores condições socioeconômicas, nos que fumam, nos que possuem acúmulos de doenças crônicas, nos que negligenciam a higiene bucal e nos que não fazem uso de próteses dentárias. Reconhece-se que esses fatores explicam apenas parte da variação observada na dificuldade alimentar, pois outros determinantes também devem ser considerados nas estratégias de intervenção e políticas públicas voltadas para a saúde dos idosos.

## Data Availability

O banco de dados, apenas com a população idosa, utilizado da Pesquisa Nacional de Saúde de 2019 está disponível em: https://drive.google.com/file/d/1VDjasU9Wrteyqfme5LmeCIAX45Z0QrXs/view?usp=sharing.
